# Inhibition of the AURKA/YAP1 axis is a promising therapeutic option for overcoming cetuximab resistance in colorectal cancer stem cells

**DOI:** 10.1038/s41416-024-02649-z

**Published:** 2024-03-11

**Authors:** Anxo Rio-Vilariño, Aiora Cenigaonandia-Campillo, Ana García-Bautista, Pedro A. Mateos-Gómez, Marina I. Schlaepfer, Laura del Puerto-Nevado, Oscar Aguilera, Laura García-García, Carlos Galeano, Irene de Miguel, Juana Serrano-López, Natalia Baños, María Jesús Fernández-Aceñero, Juan Carlos Lacal, Enzo Medico, Jesús García-Foncillas, Arancha Cebrián

**Affiliations:** 1 Translational Oncology Division, Oncohealth Institute, Instituto de Investigación Sanitaria Fundación Jiménez Díaz, Fundación Jiménez University Hospital (IIS-FJD, UAM), Madrid, Spain; 2 Biochemistry and Molecular Biology Unit, Department of System Biology, School of Medicine and Health Sciences, University of Alcalá. Alcalá de Henares, Madrid, Spain; 3 Pathology Department, IIS-Fundación Jiménez Diaz-UAM, Madrid, Spain; 4 Experimental Hematology Lab, IIS-Fundación Jimenez Díaz, UAM, Madrid, Spain; 5 Preclinical program START Madrid-FJD, Hospital Fundación Jiménez Díaz-UAM, Madrid, Spain; 6 Department of Pathology, Hospital Clínico San Carlos, Instituto de Investigación Sanitaria del Hospital Clínico San Carlos (IdISSC), Madrid, Spain; 7 Instituto de Investigaciones Biomédicas, CSIC/UAM, Madrid, Spain; 8 Instituto de Investigación Sanitaria Hospital La Paz, IDIPAZ, Madrid, Spain; 9 Department of Oncology, Università degli Studi di Torino, Candiolo (TO), Italy; 10 Candiolo Cancer Institute, FPO-IRCCS, Candiolo (TO), Italy

**Keywords:** Colorectal cancer, Cancer stem cells, Cancer therapeutic resistance

## Abstract

**Background:**

Primary resistance to anti-EGFR therapies affects 40% of metastatic colorectal cancer patients harbouring wild-type *RAS/RAF*. YAP1 activation is associated with this resistance, prompting an investigation into AURKA’s role in mediating YAP1 phosphorylation at Ser397, as observed in breast cancer.

**Methods:**

We used transcriptomic analysis along with in vitro and in vivo models of *RAS/RAF* wild-type CRC to study YAP1 Ser397 phosphorylation as a potential biomarker for cetuximab resistance. We assessed cetuximab efficacy using CCK8 proliferation assays and cell cycle analysis. Additionally, we examined the effects of AURKA inhibition with alisertib and created a dominant-negative YAP1 Ser397 mutant to assess its impact on cancer stem cell features.

**Results:**

The *RAS/RAF* wild-type CRC models exhibiting primary resistance to cetuximab prominently displayed elevated YAP1 phosphorylation at Ser397 primarily mediated by AURKA. AURKA-induced YAP1 phosphorylation was identified as a key trigger for cancer stem cell reprogramming. Consequently, we found that AURKA inhibition had the capacity to effectively restore cetuximab sensitivity and concurrently suppress the cancer stem cell phenotype.

**Conclusions:**

AURKA inhibition holds promise as a therapeutic approach to overcome cetuximab resistance in *RAS/RAF* wild-type colorectal cancer, offering a potential means to counter the development of cancer stem cell phenotypes associated with cetuximab resistance.

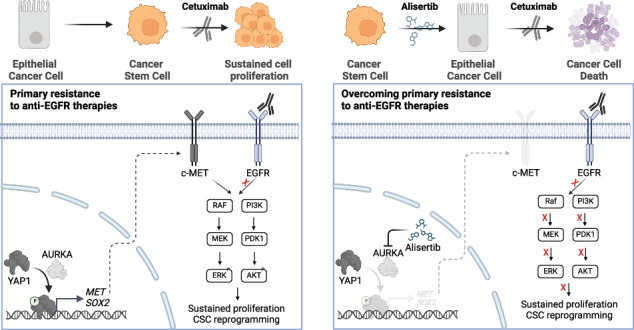

## Introduction

Colorectal cancer (CRC) is a leading cause of cancer-related deaths worldwide, with 25% of individuals being diagnosed with metastatic disease [1]. Despite advances in treatment, the survival rate for these patients remains poor. Targeted therapies against the Epidermal Growth Factor Receptor (EGFR), such as cetuximab, have been FDA-approved for the treatment of patients with *RAS/RAF* wild-type metastatic CRC (mCRC) [2]. However, 40% of them experience disease progression while suffering from adverse effects [3], highlighting the need for new biomarkers to predict response to anti-EGFR therapies.

Drug resistance is often driven by the acquisition of cancer stem cell (CSC) phenotypes in several cancers, including colon cancer [4], due to the associated self-renewal capacity, plasticity, and quiescence features. Pathways known to promote CSC traits, like Sonic Hedgehog [5], Notch [6], or Hippo [7–9] have been linked to resistance against EGFR tyrosine kinase inhibitors. Yes-Associated Protein 1 (YAP1), the main effector of the Hippo pathway, is a well-described oncogenic driver in CRC. [10–12] Despite this, the lack of YAP1-specific inhibitors renders it an undruggable therapeutic target [13]. Nonetheless, since its activity is fundamentally regulated by phosphorylation [14], targeting its upstream kinases could be a potential therapeutic alternative to control its oncogenic activity.

Recent studies have shown that nuclear phosphorylation of YAP1 at Ser397 by Aurora Kinase A (AURKA), a master cell cycle regulator [15], enhances its stabilisation and improves its transcriptional activity [16]. Specific AURKA inhibitors like alisertib (MLN8237) are available [17] and are currently under clinical evaluation for the treatment of solid tumours both in monotherapy and combination [18, 19].

While AURKA and YAP1 have been previously associated with cetuximab resistance individually, their combined role remains unstudied. In this study, we present the first evidence that there is a subgroup of mCRCs displaying increased phosphorylation of YAP1 at Ser397, resulting in augmented stem-like properties that confer resistance to cetuximab. Sublethal alisertib regimes restrict YAP1 Ser397 phosphorylation, overcome cetuximab resistance, and reverse stem-like properties by reducing c-MET levels both in vitro and in vivo. The expression of negative-dominant mutants of YAP1 lacking the Ser397 phosphorylation site prevents YAP1-induced c-MET expression and subsequent stemness properties. These findings suggest that evaluating YAP1 phosphorylation at Ser397 could serve as a biomarker for predicting cetuximab response and identifying patients who may benefit from combined therapies with AURKA inhibitors.

## Materials and Methods

### Cell culture and reagents

Human CRC cell line HCA46 was obtained from European Collection of Authenticated Cell Cultures (ECACC). SW48 and CaCo2 were obtained from the American Type Culture Collection (VA, USA). Meanwhile, C10 and KM12SM cell lines were kindly provided by Prof. Alberto Bardelli from Torino University. HEK293T cells were gifted by Dr. Pedro Mateos from the University of Alcalá. All the cell lines were authenticated by short tandem repeat (STR) profiling. HCA46, C10, KM12SM, and HEK293T cells were cultured with Dulbecco Modified Eagle’s Medium (DMEM; HyClone, UT, USA). SW48 and CaCo2 cells were cultured with Roswell Park Memorial Institute (RPMI) medium. In both cases, media were supplemented with 25 mM HEPES (GibcoTM; ref 22400_097), 10% FBS and 1% penicillin/streptomycin. All cells were maintained in cell culture flasks incubated at 37^o^C with 5% CO_2_ and controlled humidity. Routine screening for the presence of mycoplasma occurred bimonthly.

Cetuximab (Erbitux®) at 5 mg/mL was obtained from Merck KGaA©. Alisertib (MLN8237®; ref. HY-10971) was obtained from MedChemExpress® and dissolved in DMSO to obtain stocks at 1 mM. EGF was obtained from MedChem Express (HY-P7109) and dissolved in water to obtain stock at 100 μg/mL. All drugs were filtered with a 0.22 nm filter and diluted at different concentrations in DMEM or RPMI media (Fisher brand™; ref. 15206869). Stocks were kept at –20 ^o^C or -80 °C until use, following manufacturers’ instructions.

### Vector construction

To generate the vector for YAP1 overexpression, YAP1-V5 in pLX304 (Addgene #42555; http://n2t.net/addgene:42555) gifted by William Hahn was used. pLX304 plasmid was used as empty vector. YAP^S397A^ mutant form was generated via overlapping PCR together with site-directed mutagenesis using primers described in Table S1. YAP1-V5 pLX304 was used as template and PCR product cloned into pLX304 vector using BamHI and NheI sites. The presence of YAP1 S397A mutation was confirmed by sequencing.

### Transfection and lentivirus production

HEK293T cells were seeded and cultured to 90% confluence. Cells were transfected with 10 µg of lentiviral expression vector and 10.5 µg of viral packaging plasmids (3 µg of pVSVG, 5 µg of RRE and 2.5 µg of RRV) using 1 mg/ml polyethyleneimine (PEI) in a 3:1 ratio with respect to DNA quantity. Media was replaced after 8 hours post-transfection. Forty-eight hour later, the media was collected and filtered with 0.45 nm filters (Fisher brand™ ref. 15216869) and then supplemented with 10 ng/µL of polybrene (Merck H9268-5G). For infection, the viral soup was added to the SW48 and C10 cell lines and renewed every two hours up to four times. After 24 hours, Blasticidin 20 µg/mL (Sigma-Aldrich, ref. SBR00022-1ML) was added to select the transduced cell lines over a total of 120 hours.

### RNA extraction, complementary DNA synthesis (cDNA), and Quantitative PCR (qPCR)

The detailed protocol of RNA extraction and RT-qPCR was reported previously [20]. TaqMan® probes (Thermo Fisher Scientific) used for evaluating gene expression are the following: *CYR61* (Hs00155479_m1), *CTGF* (Hs00170014_m1), *SOX2* (Hs04234836_s1), *RPLP0* (Hs99999902_m1). The expression ratio was calculated by the ΔΔCt method as described by Livak and Schmittgen [21], using *RPLP0* as the housekeeping gene for data normalisation. Each sample was analysed in duplicate or triplicate.

### Western blot analysis and antibodies

Western blotting protocol was performed as previously described [20]. 20–30 µg of protein lysate was used in each experiment. Primary antibodies used for experiments are the following: ERK (Cell Signaling Technology, #4695, 1:1000), p-ERK (Cell Signaling Technology, #4370, 1:1000), AKT (Cell Signaling Technology, #4691, 1:1000), p-AKT (Santa Cruz Biotechnology, sc-7985-R, 1:250), MET (Proteintech, 25869-1-AP, 1:1000), Tubulin (Proteintech, 66031-1-Ig, 1:3000), YAP1 (Cell Signaling Technology, #14074, 1:1000), p-Ser397-YAP1(Cell Signaling Technology, #13619, 1:1000), MOB-1 (Cell Signaling Technology, #13730, 1:1000), p-MOB-1 (Cell Signaling Technology, #8699 S, 1:1000), LATS-1 (Cell Signaling Technology, #3477,1:1000), p-LATS-1 (Cell Signaling Technology, #8654, 1:1000). Anti-mouse (GE Healthcare, United Kingdom, ref. NA931V) or anti-rabbit (GE Healthcare, UK, ref. NA934V) secondary antibodies were used at 1:10000 dilution.

### Cell Viability Assays

7000 cells were seeded in 96 well plates and incubated for 24 hours at 37 °C in a humidified incubator with 5% CO_2_. The media was replaced, and cells were exposed to alisertib (350 nM for C10 and 35 nM for SW48) for 48 hours followed by 72 hours of cetuximab treatment (10 ng/µL for C10 and 1 ng/µL for SW48). The alisertib concentration was chosen to cause less than 30% of death after 72 hours of treatment. In the case of cetuximab, the minimum concentration that elicited an optimal response after combination with alisertib was selected.

To determine the survival percentage of treated cells relative to the control, Cell Counting Kit (CCK8, Sigma Aldrich #96992) was used according to the manufacturer’s instructions.

### Colony formation and colonosphere assay

C10 and SW48 cell lines were seeded in 100 mm plates to reach 70% confluence after 24 hours. Cells were treated with alisertib or cetuximab, using the doses described above. After 48 hours, cells were stained with trypan blue and counted.

For the colony formation assay, 3000 live cells were seeded in 100 mm plates. After 10 days, colonies were stained with methyl violet dye (Thermo Fischer Scientific) and counted.

For the colonosphere formation assay, 1000 viable cells were grown in special polystyrene plates (Corning) that promote the growth of ultralow adherence cells. These cells were cultured in a mixture of DMEM/F12, insulin (20 mg/ml, Sigma), EGF (20 ng/ml, R&D Systems), bFGF (10 ng/ml, R&D Systems), glucose (3 mg/ml, Sigma), and antibiotics (1% penicillin-streptomycin, PanBiotech). The plates were incubated at 37°C with 5% CO_2_ for 10 days when photographs of the spheres were taken. The size of the spheres was measured using ImageJ (NIH, Maryland, USA) [22].

### Cell cycle analysis

Cell cycle analysis of SW48 and C10 cell lines was performed using Hoescht34580 (5 µg/mL, Invitrogen) and pyronin Y (0.25 µg/mL, Sigma-Aldrich) nucleic acids dyes. Briefly, the cell pellet was fixed and permed using cytofix/cytoperm reagent (BD Bioscience) on ice for 20 min. Then, cells were washed with permeabilization buffer 1X. After being washed, cells were incubated in a pre-warmed solution containing Hoescht 34580 in a water bath at 37 °C for 30 min. Then, pyronin Y was added into the suspension cells and incubated for another 30 min. Cell acquisition was performed by flow cytometry in a FACS Canto-II machine equipped with violet, blue and red lasers (BD Biosciences). To perform the analysis, we used FACSDIVA software [23].

### Patient-derived Xenograft model

Female athymic nude mice of 5–6 weeks old from Charles River Laboratories were housed in the Animal Model Core Facility at IIS-Fundación Jiménez Díaz. All animal procedures were approved by the Ethical Animal Research Committee at IIS-Fundación Jiménez Díaz and conducted in accordance with institutional and government regulations (Reference n°: PROEXP 142-17). We analysed the *RAS/RAF* wild-type mCRC tumours available in the START Madrid-FJD preclinical biobank and selected the one with the highest levels of YAP1 Ser397 phosphorylation for our study. Fresh tumour fragments were subcutaneously implanted into the dorsal region once tumour volumes achieved an average of 200 mm^3^. Power analysis was used to calculate the sample size required for animal experiments. Mice were randomised into four branches (vehicle, alisertib, cetuximab, and combinatorial treatment) and treated for 21 days. There is no blinding of researchers or participants. Tumour growth was monitored 3 days/week by calliper measurements and tumour volume was calculated using the formula V = (width^2^ x length)/2. Additional details are provided in the Supplementary Information.

### Statistical analyses

The presented experimental data are expressed as the mean ± standard deviation (SD). The sample size (*n*) for each statistical analysis is specified in the figure captions. *P*-values (p) were determined using GraphPad Prism 5 software. Two-tailed t-Student’s test was employed for two-sample comparisons. In cases involving the comparison of three or more conditions, analysis of variance (one-way ANOVA) was initially conducted, followed by Tukey’s multiple comparisons test. If the structural assumptions of ANOVA were not met by the analysed samples, the Kruskal-Wallis test was applied. Two-way ANOVA was used to compare three or more groups when two variables were analysed. Differences were considered statistically significant then the *P* values were less than 0.05. At least three biological replicates were performed for each experiment to ensure the reliability of our results.

Other related methods were provided in the Supplementary Information.

## Results

### Cetuximab-resistant CRC cell lines exhibit elevated YAP1 activation, correlating with increased phosphorylation at Ser397

**Fig. 1:**
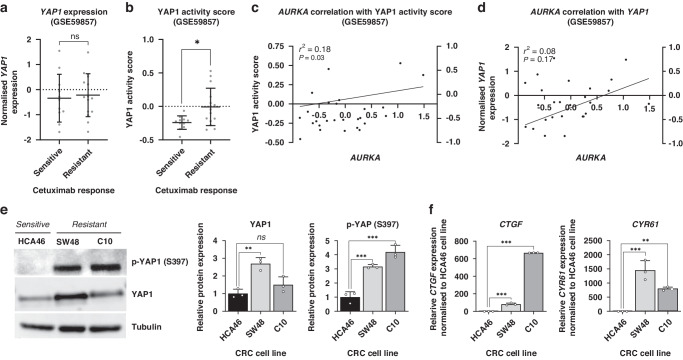
YAP1 activity correlates with Ser397 phosphorylation during primary resistance to cetuximab in CRC cell lines. **a** Plot representing the distribution of YAP1 expression in CRC cell lines sensitive to cetuximab compared to the resistant ones *ns*=non-significant *p < 0.05, two-tailed t-Student’s test. **b** Plot depicting the distribution of YAP1 activity score in cetuximab-sensitive CRC cell lines compared to the resistant ones. **c** Correlation analysis between *AURKA* expression and YAP1 activity score. **d** Correlation analysis between AURKA and YAP1 expression levels. **e** Western blot illustrating the basal levels of total amount and phosphorylated YAP1 (Ser397) in CRC cell lines HCA46, SW48 and C10. Tubulin was used as a loading control. Results are plotted as the average ± SD of all the biological replicates and were normalised to the HCA46 cell line (*n* = 3). *ns*=non-significant, **p < 0.01, ***p < 0.001, one-way ANOVA. **f** Gene expression levels of *CTGF* and *CYR61* in CRC cell lines HCA46, SW48 and C10 (*n* = 3). *ns*=non-significant, **p < 0.01, ***p < 0.001, one-way ANOVA.

To explore the potential role of YAP1 in primary resistance to cetuximab in CRC, we performed a comprehensive bioinformatic analysis using RNA microarray data [24] from CRC cell lines with known cetuximab-response status (GSE59857). Initially, we assessed the expression levels of YAP1 in intrinsically resistant and sensitive CRC cell lines and found no significant differences between the two groups (Fig. 1a). However, to gain deeper insights, we calculated a single-sample score for predicting YAP1 activation using a well-established gene signature associated with its increased transcriptional activity (more information about this signature is available online at https://www.gsea-msigdb.org/gsea/msigdb/cards/CORDENONSI_YAP_CONSERVED_SIGNATURE.html). Intriguingly, a subgroup of cetuximab-resistant cell lines displayed significantly higher YAP1 activity scores, while all the sensitive cell lines displayed low values of this score. Together, these data suggest the potential involvement of YAP1 in driving this resistant phenotype in a subset of mCRC patients (Fig. 1b).

YAP1 phosphorylation at Ser397 by AURKA has recently emerged as a notable post-translational modification that enhances its stability and transcriptional activity. However, its implications in drug resistance remain largely unexplored. To shed light on this relationship, we conducted a comprehensive linear regression analysis to examine the association of *AURKA* expression with *YAP1* expression and its activity score. Strikingly, our results revealed a significant positive correlation between *AURKA* mRNA expression and YAP1 activity score (r^2^ = 0.18, *p*-value = 0.03), indicating that AURKA likely plays an important role in modulating YAP1-driven transcriptional activity (Fig. 1c). In contrast, no significant correlation was observed between *AURKA* and *YAP1* mRNA expression levels (Fig. 1d), suggesting that AURKA primarily influences YAP1 activity rather than its overall expression in CRC cells. These findings provide further evidence for the potential involvement of the AURKA/YAP1 axis in mediating drug resistance mechanisms in CRC.

To validate our findings, we selected three *RAS/RAF* wild type CRC cell lines, including two intrinsically resistant (SW48 and C10) and one sensitive (HCA46) to cetuximab. We assessed the protein levels of YAP1 and its Ser397 phosphorylated form. Notably, the resistant cell lines exhibited a 3 and 4-fold increase in YAP1 phosphorylation compared to the sensitive HCA46 cell line. In line with prior bioinformatic analyses, these findings support heightened YAP1 activation in cetuximab-resistant phenotypes, regardless of total YAP1 expression. (Fig. 1e). To confirm the correlation between YAP1 phosphorylation and activation, we examined the expression of its main transcriptional targets, *CTGF* and *CYR61*. Remarkably, both genes showed an exacerbated expression in the resistant cell lines compared to the sensitive one, providing further support for the notion of enhanced YAP1 transcriptional activity in cetuximab-resistant phenotypes driven by Ser397 phosphorylation (Fig. 1f). These findings emphasise the potential involvement of YAP1 hyperphosphorylation in driving cetuximab resistance mechanisms in CRC.

### Inhibition of Aurora kinase A effectively overcomes cetuximab resistance by preventing the phosphorylation of YAP1 at Serine 397

**Fig. 2:**
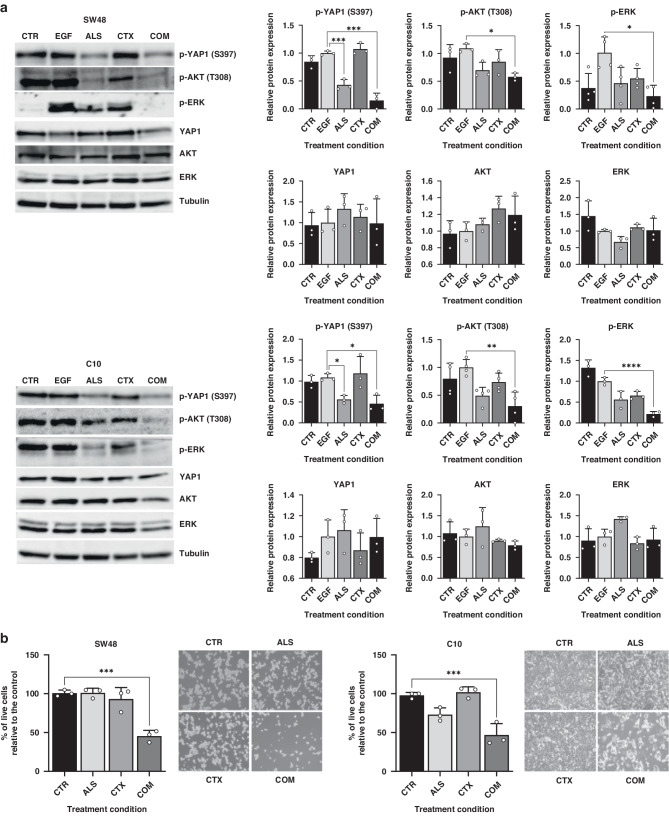
AURKA inhibitor alisertib disrupts YAP1 Ser397 phosphorylation and overcomes cetuximab resistance. **a** Western blots showing the total and phosphorylated forms of YAP1, AKT and ERK after cetuximab and/or alisertib treatment. Cells were stimulated with 40 ng/mL EGF after both treatments to induce ERK and AKT phosphorylation. Tubulin was used as loading control. Results are plotted as the average ± SD of all the biological replicates and were normalised to the EGF condition (*n* = 3, *n* = 4 for p-ERK in SW48 cell line).*ns*=non-significant, **p < 0.01, ***p < 0.001, one-way ANOVA. **b** Proliferation levels of SW48 and C10 cell lines treated with cetuximab and alisertib, either alone or in combination, relative to control (*n* = 3). *ns*=non-significant, **p < 0.01, ***p < 0.001, one-way ANOVA. CTR Control, ALS Alisertib, CTX Cetuximab, COM Combined.

Given the elevated phosphorylation of YAP1 at Ser397 observed in cetuximab-resistant CRC cell lines, we sought to verify whether this phenomenon was mediated by AURKA. Therefore, we treated the resistant cell lines with the AURKA inhibitor alisertib for 48 hours. As anticipated, notable decrease in YAP1 phosphorylation was noted, with no significant alterations in total YAP1 levels (Fig. 2a), supporting our hypothesis of AURKA-mediated control of YAP1 activation in CRC.

To assess the efficacy of targeting the AURKA/YAP1 axis to overcome cetuximab resistance, we measured the cell proliferation changes in cetuximab-resistant CRC cell lines after a combined therapy of 48 hours with alisertib followed by a 72-hour cetuximab treatment. Significantly, alisertib reinstated sensitivity to cetuximab in the resistant cell lines SW48 and C10 achieving growth reductions of more than 50% compared to non-pre-treated controls (Fig. 2b). To confirm the enhanced efficacy of cetuximab following pre-treatment with alisertib, we assessed the phosphorylation levels of the downstream kinases AKT and ERK in the EGFR pathway. These kinases are hyperphosphorylated when EGFR is active, thus increased reductions in their phosphorylated forms indicate higher efficacy of EGFR blockade achieved by cetuximab. Single AURKA inhibition led to a partial reduction in the phosphorylation levels of both ERK and AKT. However, when combined with cetuximab, the phosphorylation of both kinases was almost completely suppressed, confirming the effect of alisertib in enhancing sensitivity to cetuximab (Fig. 2a).

To corroborate that alisertib-induced reversal of cetuximab resistance stems from the inhibition of YAP1 phosphorylation at Ser397, we selected two resistant *RAS/RAF* wild type cell lines (CaCo2 and KM12SM) that exhibited low levels of YAP1 phosphorylation. As expected, AURKA inhibition failed to restore sensitivity to cetuximab treatment in these cell lines (Figure S1).

Moreover, flow cytometry-based cell cycle analysis showed that the combined treatment in cell lines with high p-Ser397 YAP1 levels exacerbated the G2/M arrest induced by alisertib monotherapy, suggesting a synergistic effect between the AURKA inhibitor and cetuximab. Notably in the C10 cell line, which displays very low proliferation rates and a quiescent behaviour, treatment with alisertib resulted in the exit of cells from G0/G1 phase and accumulation in G2/M. A further treatment with cetuximab virtually eliminated cells in the G0 phase (Figure S2).

### YAP1 phosphorylation at Ser397 promotes a cancer stem cell reprograming, favouring cetuximab resistance in CRC cells

YAP1 has been recognised as a driver of stem-like properties in CRC [9], prompting us to explore the potential involvement of YAP1 Ser397 phosphorylation in the development of primary resistance to cetuximab through the acquisition of the CSC phenotype. To investigate the relationship between CSC features and cetuximab resistance, we used 25 CSC-related signatures available in http://stemchecker.sysbiolab.eu/ and calculated their scores for each sample contained in the GSE59857 dataset. The resistant CRC cell lines showed a higher score for 23 out of the 25 signatures, being six of them statistically significant (Figure S3a). Furthermore, these six signatures showed a positive correlation with YAP1 activity (Figure S3b), suggesting that the AURKA/YAP1 axis potentially plays a role in the development of stem-like features during primary resistance to cetuximab.

**Fig. 3:**
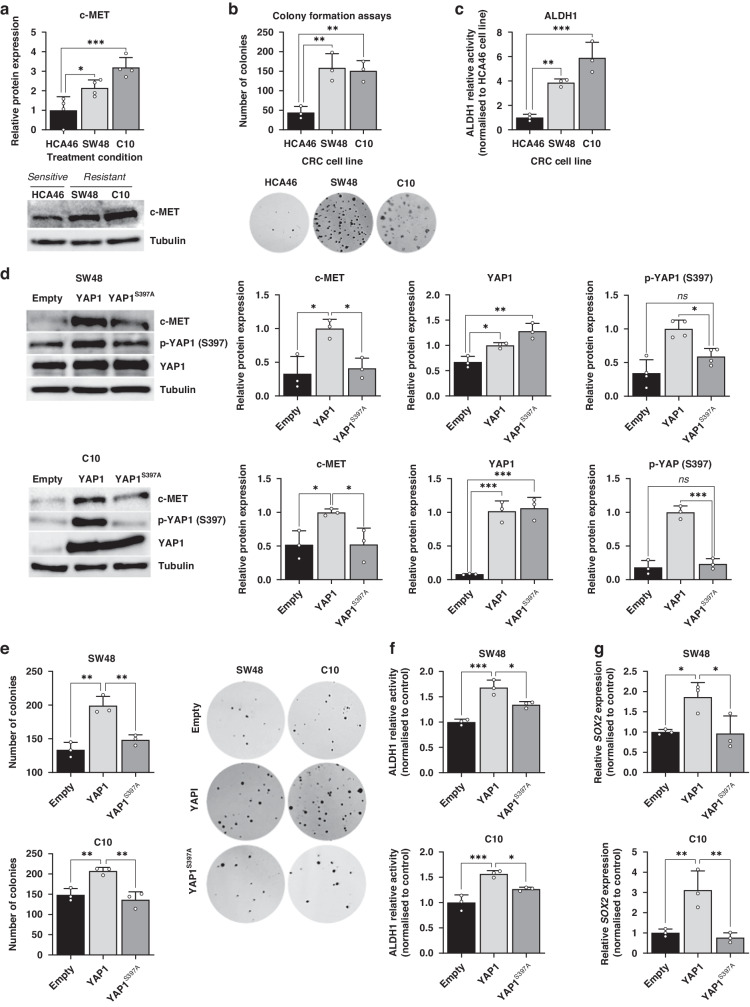
YAP1 exerts specific regulation over c-MET and CSC features through Ser397 phosphorylation. **a** Western blot illustrating c-MET levels in the HCA46, SW48 and C10 cell lines. Tubulin was used as a loading control. Results are plotted as the average ± SD of all the biological replicates and were normalized to the HCA46 cell line (*n* = 3). **b** Colony formation assay of CRC cell lines HCA46, SW48 and C10.Results are plotted as the average ± SD of all the biological replicates. **p < 0.01 (*n* = 3), one-way ANOVA. **c** ALDH1 relative activity in CRC cell lines HCA46, SW48 and C10. Results were normalized to the cetuximab-sensitive HCA46 cell line. Results are plotted as the average ± SD of all the biological replicates. **p < 0.01, ***p < 0.001, one-way ANOVA. **d** Western blot illustrating c-MET expression in SW48 and C10 cell line transduced with YAP1 and YAP1^S397A^ plasmids, along with the empty vector. Results were normalized to the YAP1-trasduced cell lines. **e** Colony formation assay of SW48 and C10 cell line transduced with YAP1 and YAP1^S397A^ plasmids, along with the empty vector. Results are plotted as the average ± SD of all the biological replicates, **p < 0.01 (*n* = 3), one-way ANOVA. **f** ALDH1 relative activity in SW48 and C10 cell line transduced with YAP1 and YAP1^S397A^ plasmids, along with the empty vector. Results were normalized to the empty vector condition in each cell line. Results are plotted as the average ± SD of all the biological replicates, *p < 0.05, **p < 0.01 (*n* = 3), one-way ANOVA. **g** *SOX2* gene expression in SW48 and C10 cell line transduced with YAP1 and YAP1^S397A^ plasmids, along with the empty vector. Results were normalized to the empty vector condition in each cell line. Results are plotted as the average ± SD of all the biological replicates *p < 0.05, **p < 0.01 (*n* = 3), one-way ANOVA.

Since it has been reported that c-MET is a transcriptional target of YAP1 and both contribute to the acquisition of stemness in CRC [9], we aimed to evaluate c-MET expression and certain CSC traits in our cell lines. We confirmed that SW48 and C10, characterised by high levels of YAP1 Ser397 phosphorylation, showed two- and three-fold higher c-MET expression, respectively, compared to the cetuximab-sensitive cell line HCA46 (Fig. 3a). Moreover, the resistant cell lines demonstrated increased stem-like properties, as evidenced by their enhanced colony-forming capacity (Fig. 3b, Figure S4) and elevated ALDH1 activity (Fig. 3c).

To assess whether the increased c-MET expression and stem-like characteristics observed in cetuximab-resistant cell lines were driven by YAP1 Ser397 phosphorylation, we generated negative dominant mutant of YAP1 lacking the Ser397 phosphorylation site (YAP1^S397A^). The overexpression of wild-type YAP1 (YAP1) led to an increase in YAP1 phosphorylation at Ser397 accompanied by a 2-to-3-fold increase in c-MET expression in both SW48 and C10 cell lines. Meanwhile, the transduction with the YAP1^S397A^ mutant led to a more than 50% reduction in c-MET levels compared to the wild type (Fig. 3d). To determine the role of YAP1 Ser397 phosphorylation in the acquisition of CSC phenotype, colony formation assays were performed. The results showed that YAP1 overexpression enhanced colony formation, whereas overexpression of the YAP1^S397A^ mutant did not (Fig. 3f, Figure S4). Additionally, an increase in ALDH1 activity was observed after overexpression of YAP1, but not of YAP^S397A^ (Fig. 3g). Finally, gene expression analysis showed an upregulation of *SOX2*, a well-established CSC marker, upon YAP1 overexpression, but not in cells transduced with the mutant vector (Fig. 3h). These findings confirm that YAP1 promotes c-MET-driven CSC reprogramming through its phosphorylation at Ser397.

### Aurora kinase A inhibition prevents stemness in CRC cell lines

**Fig. 4:**
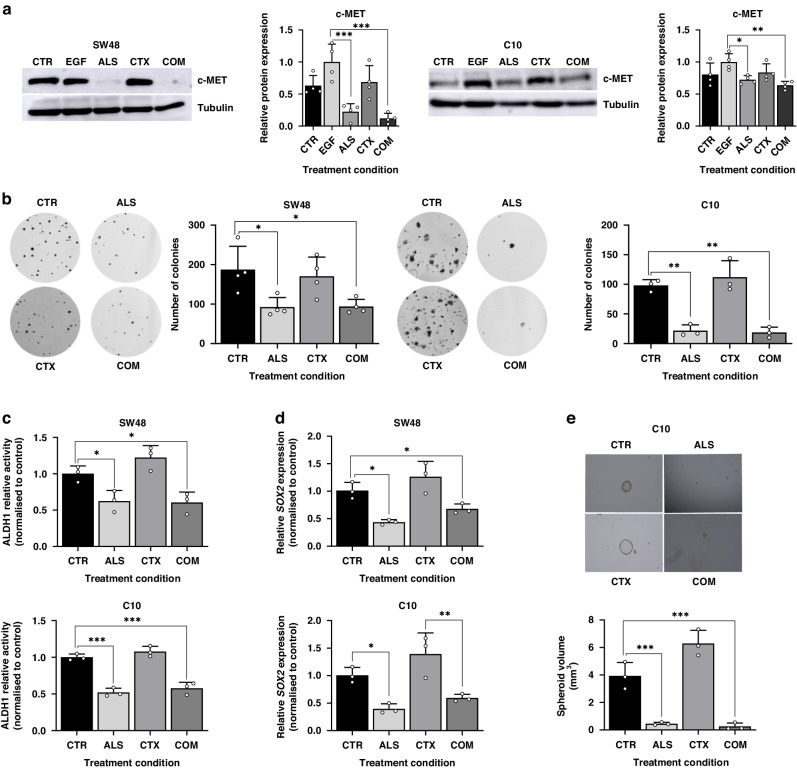
AURKA inhibition disrupts c-MET and CSC properties. **a** Western blot illustrating c-MET levels after treatment with alisertib and/or cetuximab in SW48 and C10 cell lines. Tubulin was used as loading control. Results are plotted as the average ± SD of all the biological replicates and were normalized to the EGF condition. *p < 0.05, **p < 0.01, ***p < 0.001 (*n* = 3), one-way ANOVA (*n* = 4). **b** Colony formation assay following treatment with alisertib and/or cetuximab in SW48 and C10 cell lines. Results are plotted as the average ± SD of all the biological replicates. *p < 0.05, **p < 0.01 (*n* = 3), one-way ANOVA. **c**. ALDH1 relative activity after treatment with alisertib and/or cetuximab in SW48 and C10 cell lines. Results were normalized to the control condition for each cell line and are plotted as the average± SD of all the biological replicates (*n* = 3) **p < 0.01, ***p < 0.001, one-way ANOVA. **d** *SOX2* gene expression after treatment with alisertib and/or cetuximab in SW48 and C10 cell lines. Results were normalized to the control condition in each cell line and are plotted as the average ± SD of all the biological replicates (*n* = 3). *p < 0.05, **p < 0.01 (*n* = 3), one-way ANOVA. **e**. Spheroid size (mm^3^) ability after treatment with alisertib and/or cetuximab in C10 cell line. Results are plotted as the average ± SD of all the biological replicates *p < 0.05, **p < 0.01 (*n* = 3), one-way ANOVA. CTR Control, ALS Alisertib, CTX Cetuximab, COM Combined.

These results provide support for the concept that stem-like CRC cells exhibit increased resistance to anti-EGFR therapies, and that the upregulation of c-MET orchestrated by YAP1 phosphorylation plays a significant role in this phenotype. Since YAP1 phosphorylation at Ser397 is disrupted by AURKA inhibition, we hypothesised that alisertib would suppress c-MET levels and attenuate CSC features, thus improving the response to EGFR blockade. Indeed, we observed a remarkable 73% and 37% drop in c-MET levels, respectively, in SW48 and C10 cell lines following 48-hour treatment with alisertib, both in monotherapy and in combination with cetuximab (Fig. 4a).

AURKA inhibition led to a 50% reduction in colony formation ability in the SW48 cell line, while in the C10 cell line the number of colonies was reduced by 78% with alisertib monotherapy and by 81% in combination with cetuximab (Fig. 4b, Figure S4). ALDH1 activity assays showed a 38% and 48% reduction in ALDH1 activity for SW48 and C10, respectively (Fig. 4c). Additionally, while cetuximab monotherapy showed a tendency to increase *SOX2* expression, treatment with alisertib significantly reduced the expression of this gene (around 50%) and prevented the increase in expression induced by EGFR blockade (Fig. 4d). Finally, colonosphere formation ability was evaluated in the C10 cell line after treatment with both drugs, alone and in combination. As expected, cetuximab strongly induced spheroid formation compared to the control, while treatments including alisertib inhibited colonosphere development (Fig. 4e). Together, these results indicate that AURKA inhibition prevents c-MET expression by avoiding YAP1 phosphorylation at Ser397, thereby disrupting CSC-like features and overcoming primary resistance to cetuximab in CRC.

### Targeting Aurora Kinase A restores cetuximab sensitivity in a PDX model with high YAP1 Ser397 phosphorylation

**Fig. 5:**
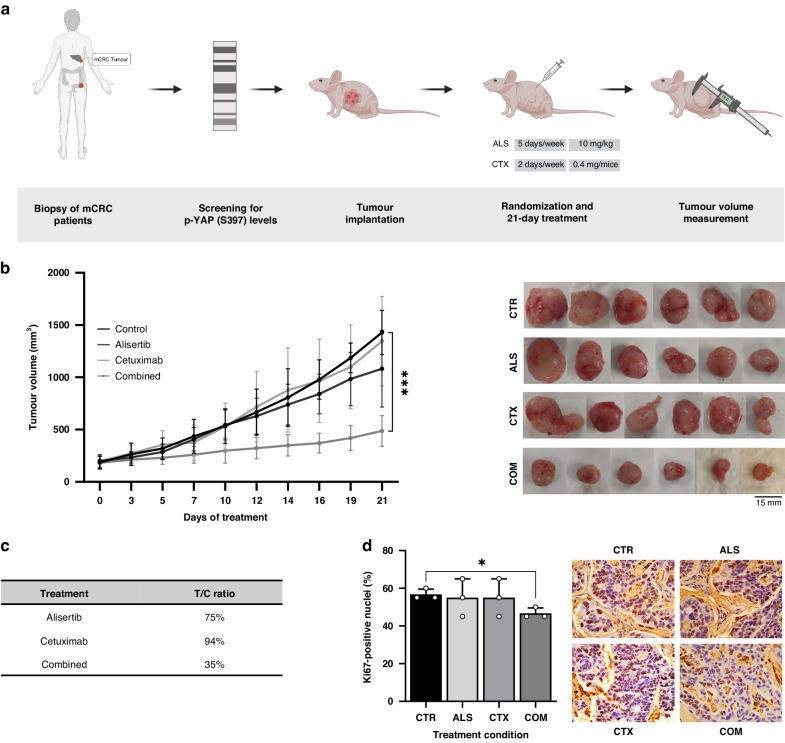
Alisertib effectively overcomes cetuximab resistance in PDX models with high levles of YAP1 phosphorylation. **a** Schematic representation outlining the criteria for selecting the tumor for the PDX model based on Western blot screening of YAP1 Ser397 phosphorylation. The tumor with the highest YAP1 phosphorylation levels was chosen for implantation. Mice were treated with 10 mg/kg/day alisertib five days a week and/or 0.4 mg/mice/day cetuximab twice a week for 21 days. Tumor volume and body weight was measured three times a week. Created with BioRender.com **b** Tumor volume measurement during 21-day treatment with alisertib and/or cetuximab. Representative images of tumors extrated from each group of mice. Results are plotted as the average ± SD of all the tumor volumes for each condition, ***p < 0.001 (*n* = 6), two-way ANOVA. **c** T/C (treated/control) ratio of tumors after the 21-day treatment with alisertib, cetuximab and combination. **d** Ki67 staining of tumors treated with alisertib, cetuximab, the combination of both or neither. *p < 0.05 (*n* = 3), Welch and Brown-Forsythe ANOVA. CTR Control, ALS Alisertib, CTX Cetuximab, COM Combined.

To evaluate the therapeutic potential of AURKA inhibition in cetuximab-resistant patients with elevated levels of YAP1 phosphorylation at Ser397, a PDX model was established as described in Fig. 5a. At the end of the experiment, tumour growth and T/C ratio were calculated. As expected, cetuximab monotherapy had no impact on tumour growth compared to the control group. Alisertib treatment showed modest antitumor activity, consistent with our in vitro analyses. However, the tumour volume of the mice treated with the combination therapy remained at 487 mm^3^, while the control group reached 1500 mm^3^ (Fig. 5b). Alisertib and cetuximab monotherapies displayed T/C ratios of 76% and 94%, respectively, while under the combination therapy it decreased to 35%, confirming the re-sensitising effect exerted by alisertib in the presence of cetuximab (Fig. 5c). Following the combined therapy, tumours exhibited a reduction of over 20% of Ki-67-positive cells compared to both the control and the monotherapy groups. However, statistically significant differences were only observed compared to the control group, likely due to the limited sample size (Fig. 5d).

**Fig. 6:**
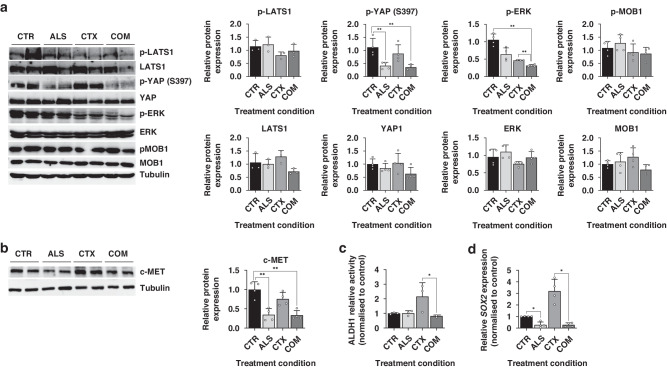
Alisertib reduces c-MET expression levels and impairs CSC features in vivo. **a** Western blot analysis of total (*n* = 3) and phosphorylated (*n* = 4) levels of LATS-1, YAP1, ERK and MOB-1 in tumors treated with alisertib and/or cetuximab, normalized to the control. Results are plotted as the average ± SD of all the biological replicates, **p < 0.01, one-way ANOVA. Differences in p-ERK expression were compared by using Welch and Brown-Forsythe ANOVA since residuals did not meet the homoscedasticity criteria of one-way ANOVA. **b** Analysis of *SOX2* expression levels in tumors after treatment with alisertib and/or cetuximab, normalized to non-treated tumors. Results are plotted as the average ± SD of all the biological replicates, *p < 0.05 (*n* = 4), one-way ANOVA. **c** Assessment of ALDH1 activity after treatment with alisertib and/or cetuximab, normalized to non-treated tumors. Results are plotted as the average ± SD of all the biological replicates, *p < 0.05 (*n* = 4), one-way ANOVA. **d**. Evaluation of the gene expression levels of *SOX2* in tumors after being treated with alisertib and/or cetuximab. Results are plotted as the average ± SD of all the biological replicates, *p < 0.05 (n = 4), one-way ANOVA.  CTR Control, ALS Alisertib, CTX Cetuximab, COM Combined.

To confirm the anti-tumour impact of combining cetuximab and alisertib, we assessed the phosphorylation levels of ERK. As expected, cetuximab monotherapy led to a partial reduction in ERK phosphorylation. However, this inhibition significantly increased when combined with the alisertib regime, demonstrating the synergistic effect of AURKA inhibition in PDX models with hyperphosphorylation of YAP1 at Ser397 (Fig. 6a).

### AURKA inhibition disrupts YAP1 Ser397 phosphorylation in vivo in a Hippo-independent manner

To verify the disruption of YAP1 activation by AURKA inhibition in vivo, we examined both the total protein levels of YAP1 and its phosphorylation at Ser397. As expected, AURKA inhibition resulted in a reduction of YAP1 phosphorylation by more than 50%, both when administered as monotherapy and in combination with cetuximab. Concurrently, the total abundance of YAP1 slightly decreased in the combined therapy, although this change did not reach statistical significance (Fig. 6a).

Given these findings, we aimed to determine whether the disruption of YAP1 Ser397 phosphorylation operates independently of the Hippo pathway, thereby supporting its activating role in vivo. We examined the key effectors of the Hippo cascade, LATS-1 and MOB-1, along with their activating phosphorylation states. These proteins, when active, play a crucial role in directing YAP1 for cytoplasmic retention and degradation. Our results indicate that both the total and phosphorylated levels of LATS-1 and MOB-1 remained largely unchanged across all treatments (Fig. 6a). Collectively, these findings establish that the reduction in YAP1 phosphorylation exerts an inhibitory effect and is contingent on AURKA activity.

### AURKA inhibition reduces CRC stemness features in vivo

To confirm the inhibitory effect of AURKA-mediated inhibition of YAP1 in CSC properties in vivo, we analysed c-MET protein levels in the PDX model. Our results showed that treatment with alisertib alone led to a 45% decrease in c-MET levels, which further decreased to 57% when combined with cetuximab (Fig. 6b). To validate the attenuation of CSC properties, ALDH1 activity (Fig. 6c) and *SOX2* expression (Fig. 6d) were evaluated. Consistently, tumours treated with alisertib, either alone or in combination, displayed over 70% reduction in *SOX2* expression compared to the control group. Notably, cetuximab-treated tumours displayed a threefold increase in *SOX-2* expression. Additionally, cetuximab treatment induced ALDH1 activity, suggesting that long-term cetuximab regimens enhance CSC-like properties. Importantly, the addition of alisertib to cetuximab prevented the increase in ALDH1 activity associated with monotherapy. Overall, these results confirm our in vitro models and demonstrate that AURKA inhibition disrupts the YAP1-driven c-MET mediated CSC phenotype in anti-EGFR therapy.

## Discussion

Predicting response to anti-EGFR therapies presents a significant challenge in managing *RAS/RAF* wild-type mCRC patients. To address it, we identify high levels of YAP1 phosphorylation at Ser397 as a potential predictor of cetuximab resistance. Our results reveal the AURKA/YAP1 axis as a therapeutic target to restore cetuximab sensitivity in preclinical models of primary resistance in mCRC. AURKA-mediated phosphorylation of YAP1 at Ser397 stabilises and activates YAP1, leading to c-MET-mediated reprogramming that promotes stemness and cetuximab resistance in mCRC.

Our bioinformatic analyses demonstrate increased YAP1 activity in cetuximab-resistant CRC cell lines, correlated with AURKA expression. In vitro, validation experiments show that cetuximab-resistant cell lines display heightened YAP1’s Ser397 phosphorylation, correlating with increased YAP1 activity, and suggesting its potential as a predictive biomarker (Fig. 1). While YAP1 phosphorylation at Ser397 is frequently associated with its targeting for cytoplasmic retention and proteasomal degradation in a Hippo-dependent manner [25–27], its role in the context of cancer remains ambiguous. Recent publications have presented evidence supporting its activating role. For example, research conducted in breast cancer shows that AURKA-mediated nuclear phosphorylation of YAP1 at this residue increases its stability and transcriptional activity [16]. Additionally, YAP1 phosphorylation by CDK7 at S397 and S127 residues, classically linked to YAP1 suppression, enhances its transcriptional activity in oesophageal squamous cell carcinoma in a Hippo-pathway independent manner [28].

We have demonstrated that AURKA promotes activating phosphorylation of YAP1 at Ser397, leading to the primary cetuximab-resistant phenotype in preclinical models of *RAS/RAF* wild-type CRC. The AURKA-specific inhibitor alisertib, currently under clinical evaluation for various cancer types, suppress YAP1 Ser397 phosphorylation and restores cetuximab sensitivity both in vitro and in PDX models, further emphasising the significance of this event in primary resistance to cetuximab (Figs. 3,  5, 6). We observed in tumour samples that AURKA inhibition does not influence the expression and phosphorylation levels of LATS-1 and MOB-1, main effectors of the Hippo-dependent YAP1 phosphorylation at Ser397 (Fig. 6a). It suggests that the reduction in YAP1 phosphorylation is independent of the Hippo kinase cascade and supports its activating role in mCRC. It is consistent with published evidence regarding the relationship between AURKA and YAP1. Wang and colleagues illustrated that AURKA stabilises YAP1 and enhances its protein levels in breast cancer models [29]. Similarly, it has been shown that TPX2, an activator of AURKA, can induce the stabilisation of YAP1 in human embryonic stem cells [30]. Without assessing the phosphorylation of YAP1 at Ser397, the authors demonstrate that inhibiting AURKA alone is sufficient to destabilise YAP1 independently from the Hippo pathway. Therefore, our findings expand AURKA’s non-mitotic functions, uncovering novel YAP1 checkpoints beyond the Hippo pathway.

CSCs are recognised for their resistance to conventional chemotherapeutics used for CRC treatment, such as 5-FU [31] or oxaliplatin [32]. Additionally, the loss of epithelial characteristics has been associated with a lack of response to cetuximab [33], and populations of CSCs resistant to anti-EGFR therapies have been identified in HNSCC [34]. YAP1 is a well-known driver of stemness features [9, 35–37]. Indeed, recent evidence has demonstrated its role in driving the acquisition of CSC traits in CRC through the induction of c-MET expression [9]. In our study, we show that the suppression of YAP1 Ser397 phosphorylation abrogates the YAP1-mediated CSC reprogramming driven by c-MET induction **(**Fig. 3**)**. Accordingly, inhibition of AURKA reduced c-MET protein levels and disrupted the CSC phenotype both in vitro and in vivo (Figs. 4,  6). These results are consistent with the decrease in ERK and AKT phosphorylation observed in cell lines treated with alisertib monotherapy (Fig. 3), since they are downstream effectors of the c-MET signalling. Thus, this tyrosine-kinase receptor converges with EGFR and its overactivation mimics that of EGFR, being it a well-documented mechanism for bypassing its blockade and promoting cetuximab resistance across various cancer types, including HNSCC, NSCLC, or CRC [38–40].

Furthermore, the increased quiescence of CSCs contributes to therapy resistance, as most cytotoxic and cytostatic agents primarily target proliferating cells [41]. YAP1 and Hippo Pathway have been linked to CSC quiescence through the loss of *MYC* expression [42], a gene significantly downregulated during primary resistance to cetuximab (data not shown). Cell cycle analyses (Figure S2) reveal that the C10 line is highly quiescent, with over half of the population in the G0/G1 phase. The combination of alisertib and cetuximab nearly eliminates cells in G0 and decreases G1 arrest by almost half. Persistent cells after EGFR inhibition have been reported to exhibit quiescent phenotypes favoured by constitutive AKT and ERK activation [43, 44]. In our model, AURKA inhibition partially supress both ERK and AKT phosphorylation, potentially reversing quiescent states that promote innate resistance to EGFR blockade.

Recent studies have shed light on the significant roles of AURKA and YAP1 in the landscape of cetuximab resistance. While investigations on the combined efficacy of AURKA inhibitors and cetuximab in vivo are limited to a single report, the results have revealed diverse outcomes, ranging from synergistic to antagonistic effects [45]. These findings highlight that the potential of AURKA inhibition in combination with cetuximab is limited to a subset of CRCs, supporting the notion that YAP1 hyperphosphorylation at Ser397 would determine the success of this therapeutic strategy (Figure S1). YAP1 activation has been associated with poor prognosis in *RAS* wild-type patients and heightened resistance to anti-EGFR therapies [7]. Furthermore, suppressing YAP1 expression has shown the potential to enhance cetuximab efficacy at higher doses in *KRAS* mutant cell lines [8]. However, there is still much to uncover regarding the molecular mechanisms underlying YAP1-mediated resistance in *RAS/RAF* wild-type CRC. In our study, we have unravelled the puzzle of AURKA-mediated cetuximab resistance, highlighting YAP1 phosphorylation by AURKA as a promising predictive biomarker for cetuximab response in mCRC. Moreover, our investigation has shown previously unrecognised mechanisms involving Hippo-independent YAP1 phosphorylation at Ser397 and the subsequent activation of stem-cell transcriptional programs as critical drivers of targeted therapy resistance. Future studies should focus on exploring other specific transcriptional programs mediated by YAP1 Ser397 phosphorylation to elucidate the full extent of this post-translational modification’s role in drug resistance.

### Supplementary information

Supplementary_Information
