# Correction: Inhibition of the AURKA/YAP1 axis is a promising therapeutic option for overcoming cetuximab resistance in colorectal cancer stem cells

**DOI:** 10.1038/s41416-024-02667-x

**Published:** 2024-04-04

**Authors:** Anxo Rio-Vilariño, Aiora Cenigaonandia-Campillo, Ana García-Bautista, Pedro A. Mateos-Gómez, Marina I. Schlaepfer, Laura del Puerto-Nevado, Oscar Aguilera, Laura García-García, Carlos Galeano, Irene de Miguel, Juana Serrano-López, Natalia Baños, María Jesús Fernández-Aceñero, Juan Carlos Lacal, Enzo Medico, Jesús García-Foncillas, Arancha Cebrián

**Affiliations:** 1 Translational Oncology Division, Oncohealth Institute, Instituto de Investigación Sanitaria Fundación Jiménez Díaz, Fundación Jiménez University Hospital (IIS-FJD, UAM), Madrid, Spain; 2 Biochemistry and Molecular Biology Unit, Department of System Biology, School of Medicine and Health Sciences, University of Alcalá. Alcalá de Henares, Madrid, Spain; 3 Pathology Department, IIS-Fundación Jiménez Diaz-UAM, Madrid, Spain; 4 Experimental Hematology Lab, IIS-Fundación Jimenez Díaz, UAM, Madrid, Spain; 5 Preclinical program START Madrid-FJD, Hospital Fundación Jiménez Díaz-UAM, Madrid, Spain; 6 Department of Pathology, Hospital Clínico San Carlos, Instituto de Investigación Sanitaria del Hospital Clínico San Carlos (IdISSC), Madrid, Spain; 7 Instituto de Investigaciones Biomédicas, CSIC/UAM, Madrid, Spain; 8 Instituto de Investigación Sanitaria Hospital La Paz, IDIPAZ, Madrid, Spain; 9 Department of Oncology, Università degli Studi di Torino, Candiolo (TO), Italy; 10 Candiolo Cancer Institute, FPO-IRCCS, Candiolo (TO), Italy

**Keywords:** Colorectal cancer, Cancer stem cells, Cancer therapeutic resistance

Correction to: *British Journal of Cancer* 10.1038/s41416-024-02649-z, published online 11 March 2024

In this article, the funding section needs be rephrased. Currently it reads:

“This work was supported by the grant PI19/01231 funded by AEI and the European Union. A.R-V contract was funded by the program “CONTRATOS PREDOCTORALES DE FORMACIÓN EN INVESTIGACIÓN EN SALUD (PFIS)”, grant FI20/00213 from the Instituto de Salud Carlos III (ISC-III) and co-funded by the European Union associated with the project PI19/01231.”

It should be updated to:

“This work was supported by the grant PI19/01231 funded by AEI and the European Union. A.R-V contract was funded by the program “CONTRATOS PREDOCTORALES DE FORMACIÓN EN INVESTIGACIÓN EN SALUD (PFIS)”, grant FI20/00213 from the Instituto de Salud Carlos III (ISC-III) and co-funded by the European Union (ERDF/ESF, “A way to make Europe”/“Investing in your future”) associated with the project PI19/01231.

The original article has been updated.

