# Supplementary material for: Inhibition of the AURKA/YAP1 axis is a promising therapeutic option for overcoming cetuximab resistance in colorectal cancer stem cells
Source: Br J Cancer. 2024 Mar 11;130(8):1402–13. doi: 10.1038/s41416-024-02649-z (PMC11014903; doi:10.1038/s41416-024-02649-z)
Supplement: Supplementary file 1 — Supplementary_Information [file 41416_2024_2649_MOESM1_ESM.doc]

**Supplementary Information.**

This supplemental file contains additional methodological details that couldn't be accommodated in the main text due to space constraints. It also includes supplementary figures for reference.

**1. Supplementary Materials and Methods.**

**Bioinformatic analysis of publicly available transcriptomic datasets.**

Gene expression data from 157 CRC cell lines were obtained from Gene Expression Omnibus (GEO) database under the identifier GSE59857. To closely reflect the clinical context of anti-EGFR treatments, samples harboring activating mutations in *RAS* and *BRAF* were excluded from the analysis. Moreover, only samples exhibiting extreme response phenotypes to anti-EGFR treatment (extremely sensitive and highly resistant) were included in our analysis.

The cell lines COLO320 and its derivates, COLO320HSR and COLO320DM (GSM1448152, GSM1448173, GSM1448182), were excluded from the analysis due to their neuroendocrine features. Additionally, the cell line HuTu80 (GSM1448180) was also excluded as it originates from the small intestine, leading to a distinct expression profile compared to colorectal-derived cell lines.

The intensity data of the microarray experiments available in GEO were obtained using the “getGEO” function of the GEOquery package. A gene signature for YAP1 activation (available at: https://www.gsea-msigdb.org/gsea/msigdb/cards/CORDENONSI_YAP_CONSERVED_SIGNATURE.html) and 25 CSC-related signatures (publicly available athttp://stemchecker.sysbiolab.eu/) were used to calculate a score for each sample. For each sample, the gene signature score was calculated as the sum of the normalized gene expression for each gene in the signature divided by the number of genes detected by the gene expression microarray. Samples were divided into two groups according to its cetuximab responsiveness status (sensitive or resistant) and differences for each signature were calculated using a two-tailed student’s t-test. For correlation analysis between gene signatures or gene expressions, Pearson’s linear regression model was used.

**Use of Large Language Models (LLMs).**

ChatGPT, an AI-based language model, was used as a tool to assist in improving the grammar and language quality of this work. All the text generated by ChatGPT was based on content previously written by the authors, and its final version was subsequently reviewed.

**Aldehyde dehydrogenase activity assay.**

ALDH1 activity detection kit (Cat # MAK082, Sigma) was used to determine the ALDH1 activity according to the recommended procedure from manufacturers.

**Immunohistochemistry.**

Tissue sections (4 μm thick) were obtained from formalin-fixed and paraffin-embedded tumours. Antigen retrieval was performed using PT-Link (Dako, Glostrup, Denmark) for 20 min at 95 °C in Citrate buffer pH 6 (Master Diagnostica). Endogenous peroxidase was blocked by immersing the sections in 0.03% hydrogen peroxide for 5 min. Slides were washed for 5 min with Tris-buffered saline solution containing Tween 20 at pH 7.6 and incubated with the primary antibody (Ki67, Dako) for 1 h at room temperature, followed by 30 min incubation with anti-Ig horseradish peroxidase-conjugated polymer (EnVision, Dako). Sections were then visualized with 3,3′-diaminobenzidine as a chromogen and counterstained with hematoxylin.

**2. Supplementary Figure Legends.**

**Figure S1. Proliferation assays of CaCo2 and KM12SM cells.** Cells were subjected to a 48-hour pre-treatment with Alisertib (800 nm and 250 nM, respectively) or DMSO (control) followed by a 72-hour treatment with cetuximab (10 ng/uL). CTR = Control, ALS = Alisertib, CTX = Cetuximab, COM = Combined.

**Figure S2. Cell cycle analysis of SW48 and C10 cell lines following a 48-hour treatment with alisertib and/or cetuximab.** Scatter plots, sections are named from P2 to P7 as following: P2 = G0, P3 = G1, P4 = S, P5 = G2, P6 = DNA complexity / Aneuploid Cells; P7 = subG0 / Apoptotic Cells. CTR = Control, ALS = Alisertib, CTX = Cetuximab, COM = Combined.

**Figure S3. Bioinformatic analysis of GSE59857 predicts resistant cells to display increased stemness features. A**. Analysis of 25 publicly available gene signatures associated with CSC features revealed that 23 of them exhibited high expression levels in cetuximab-resistant samples. Among these signatures, six achieved statistically significant differences. **B**. All the signatures with significant differences between sensitive and resistant samples exhibited a positive correlation with YAP1 Activity.

**Figure S4. Original images of colony formation assays of SW48 and CRC cell lines presented in the manuscript.** CTR = Control, ALS = Alisertib, CTX = Cetuximab, COM = Combined.

**3. Supplementary Figures.**

**Figure S1.**

**Figure. S2.**

**
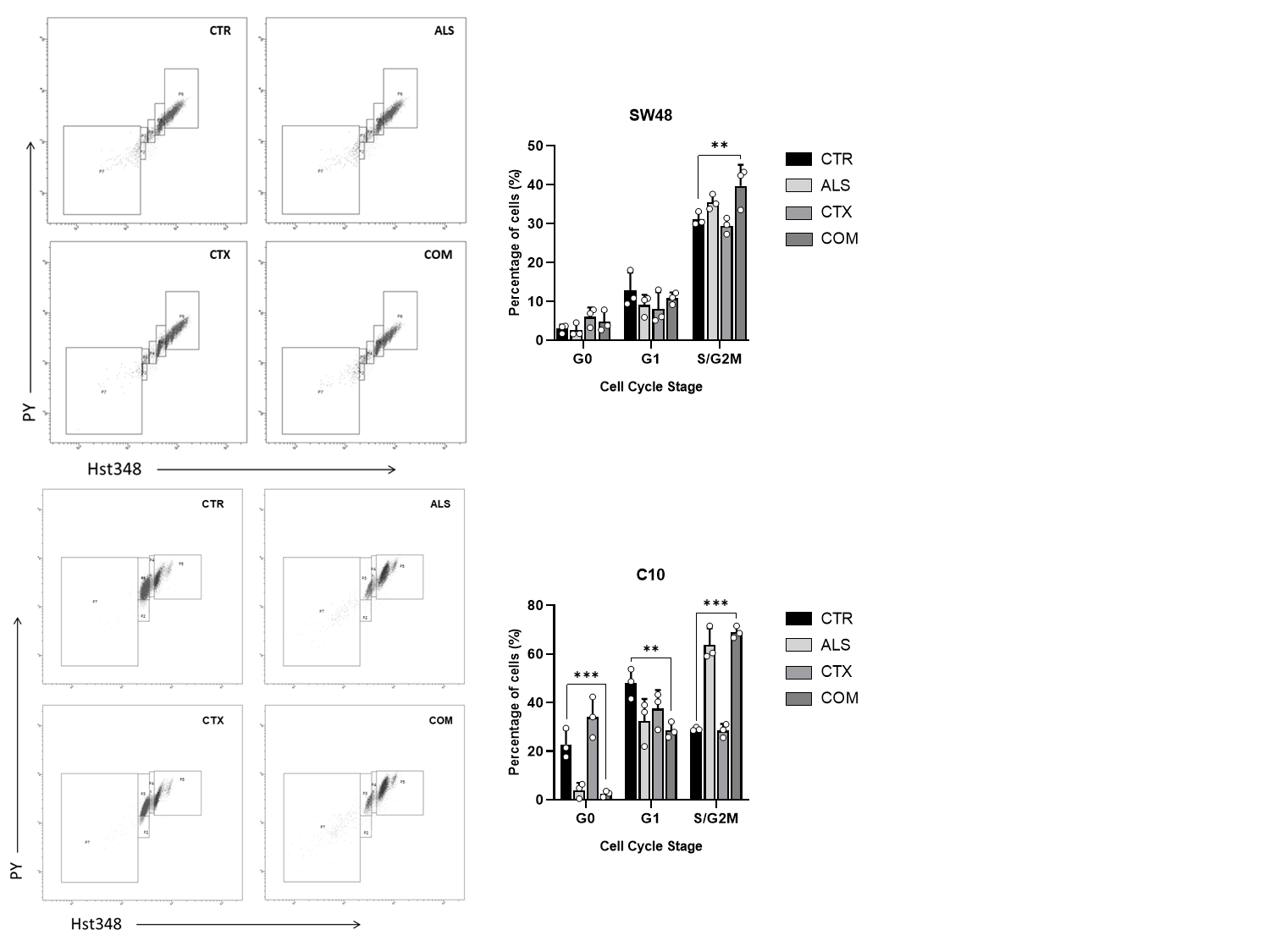
**

**Figure S3.**


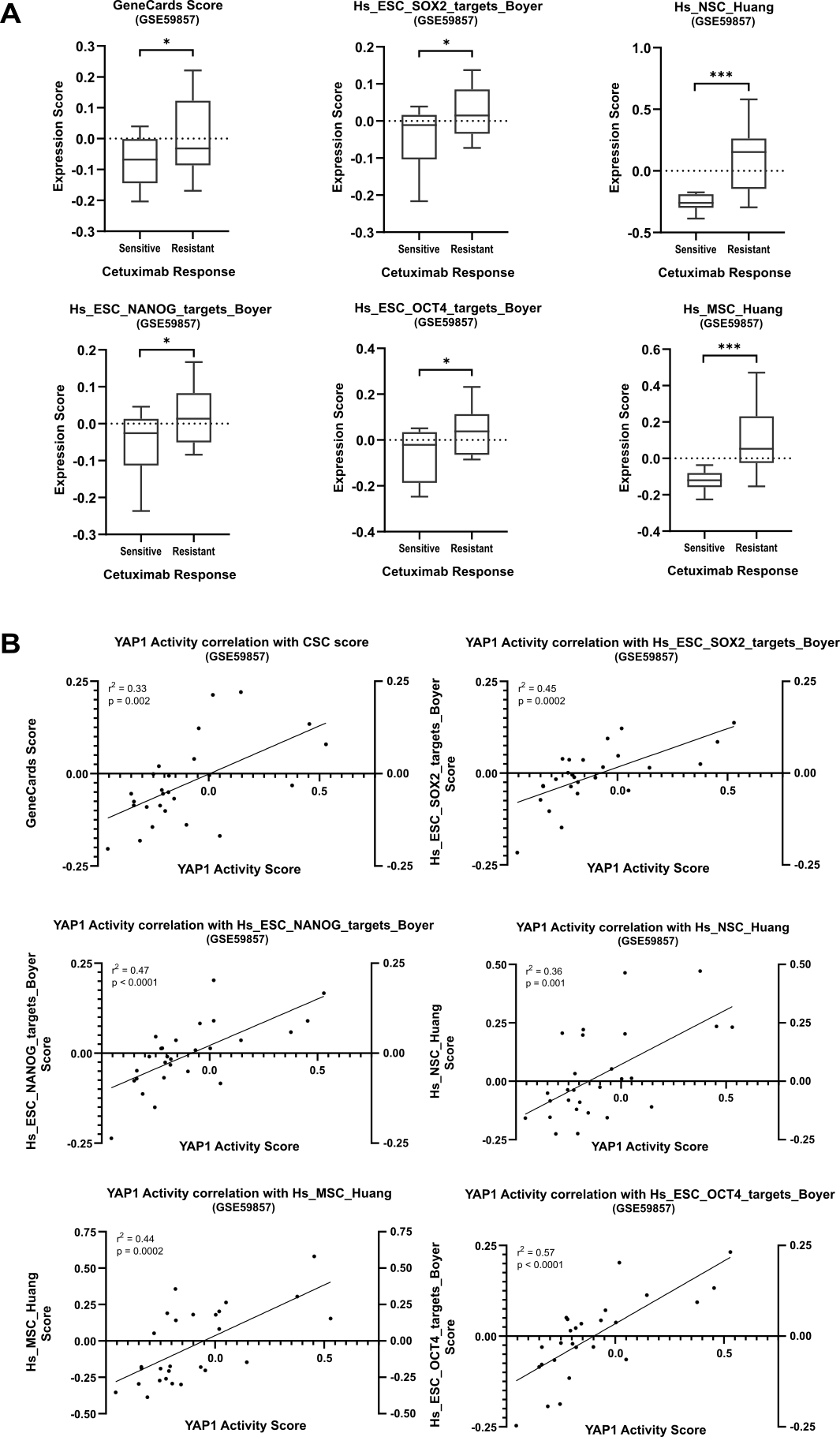


**Figure S4**

**
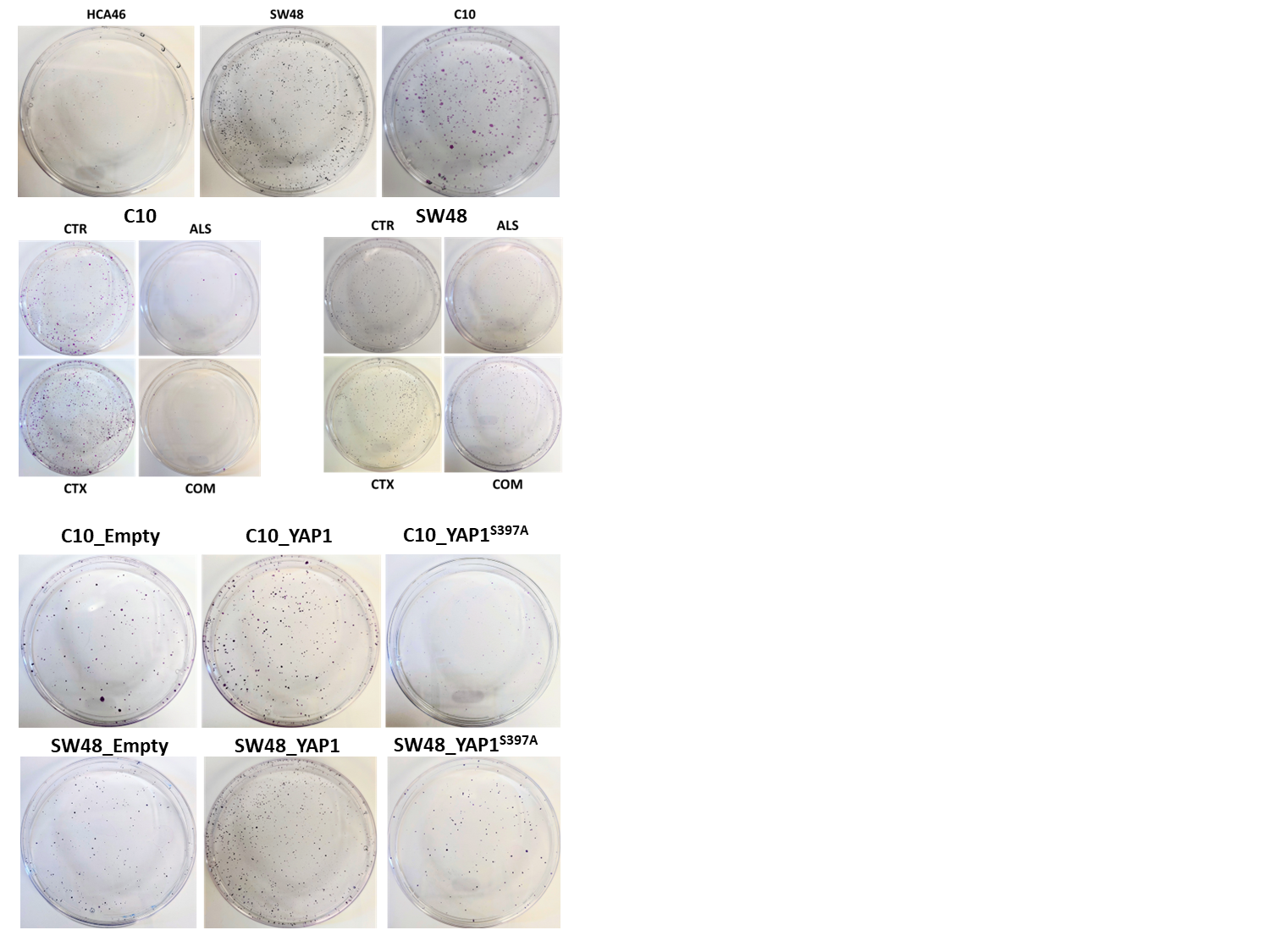
**

**4. Supplementary Tables.**

**Table S1.** Sequences of primers used for PCR-mediated site-directed mutagenesis of YAP1 contained plasmid.

| **Forward primer** | **catGGATCCcgggcagcag** |
| --- | --- |
| **Reverse primer** | **agcGCTAGCtcattactacgtag** |
| **Forward overlapping primer** | **cactctcgagatgagGCtacagacagtggactaagc** |
| **Reverse overlapping primer** | **gcttagtccactgtctgtaGCctcatctcgagagtg** |
